# Pathophysiology, Biomarkers, and Therapeutic Modalities Associated with Skeletal Muscle Loss Following Spinal Cord Injury

**DOI:** 10.3390/brainsci10120933

**Published:** 2020-12-02

**Authors:** Kelsey P. Drasites, Ramsha Shams, Vandana Zaman, Denise Matzelle, Donald C. Shields, Dena P. Garner, Christopher J. Sole, Azizul Haque, Narendra L. Banik

**Affiliations:** 1 Department of Neurosurgery, Medical University of South Carolina, 96 Jonathan Lucas St., Charleston, SC 29425, USA; drasites@musc.edu (K.P.D.); shams@musc.edu (R.S.); zamanv@musc.edu (V.Z.); matzeldd@musc.edu (D.M.); donshields@sbcglobal.net (D.C.S.); 2 Department of Microbiology and Immunology, Medical University of South Carolina, 173 Ashley Avenue, Charleston, SC 29425, USA; 3 Department of Health and Human Performance, The Citadel, 171 Moultrie St, Charleston, SC 29409, USA; garnerd1@citadel.edu (D.P.G.); csole@citadel.edu (C.J.S.); 4 Ralph H. Johnson Veterans Administration Medical Center, 109 Bee St, Charleston, SC 29401, USA

**Keywords:** muscle atrophy, calpain, inflammation, estrogen, biomarkers, neuroprotection

## Abstract

A spinal cord injury (SCI) may lead to loss of strength, sensation, locomotion and other body functions distal to the lesion site. Individuals with SCI also develop secondary conditions due to the lack of skeletal muscle activity. As SCI case numbers increase, recent studies have attempted to determine the best options to salvage affected musculature before it is lost. These approaches include pharmacotherapeutic options, immunosuppressants, physical activity or a combination thereof. Associated biomarkers are increasingly used to determine if these treatments aid in the protection and reconstruction of affected musculature.

## 1. Introduction

Recent studies reported that between 250,000 and 368,000 individuals in the United States are affected by spinal cord injuries (SCI); 17% of these individuals are veterans [1]. There are approximately 17,800 new cases documented annually [2]. This number has risen by 5000 over the past few years and has created a large financial handicap for many families [3]. This injury brings numerous secondary complications, including diabetes, cardiovascular disease, and bone demineralization, which further contribute to the financial hardship [4,5,6]. While both males and females are affected by SCI, women are found to have greater improvements in overall recovery, possibly due to the beneficial effects of estrogen [7,8].

The dexterity and range of SCI specific to secondary symptoms are related to the individual injury severity and spinal cord level [3,5,9,10]. Secondary acute changes in the spinal cord appear seconds to minutes after primary injury, with irreversible damage to neurons/glia at the injury site due to vascular disruption, free radical release, calcium influx, excitotoxicity, and lipid peroxidation. The acute phase can also be characterized by increased numbers of astrocytes/microglia and infiltration of peripheral lymphocytes, macrophages, and neutrophils. Secondary subacute alterations continue with apoptotic cell death [11], chondroitin sulphate proteoglycan (CSPG) production [12], axonal demyelination [13], and the development of glial scarring adjacent to the injury site [14]. The chronic phase changes over months/years following injury, including cystic cavity formation and maturation of the glial scar [9,15].

The chronic phase is notable for the onset of muscle atrophy occurring distal to the lesion site, due to a decrease in contractile forces placed on the joints after extended periods [9,16]. Musculoskeletal atrophy is associated with a decrease in bone mineral density, an increase in adipose tissue, and damage to peripheral nerve axons and mitochondria [9,17,18,19,20]. Thus, strategies to reduce the extent of muscle atrophy following SCI employ physical exercise, often with the combination of pharmacological treatments [7,18]. Exercise alone has been proven to stimulate muscle fiber regeneration, whereas pharmacological agents aid in the regeneration of axons, increase mitochondrial activity, and improve locomotor function [17,19]. Therefore, combinations of physical activity and pharmacological modalities may allow for the greatest improvement in functional outcomes.

## 2. Muscle Atrophy

**Figure 1:**
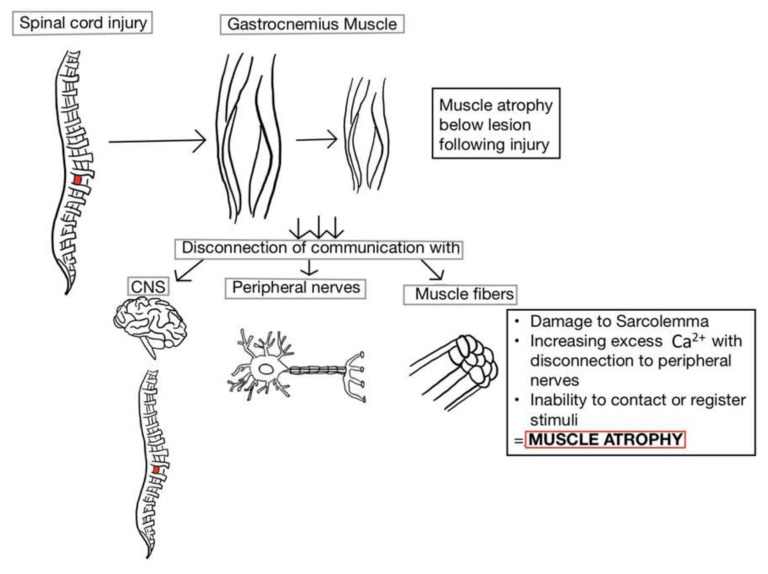
The breakdown of skeletal muscle occurs distal to the lesion site following spinal cord injury (SCI). Due to disconnection between the peripheral nerves and associated muscle fibers, the impaired sarcolemma prevents the retention of excess Ca^2+^ from being secreted. Although there is a resulting increase in the number of action potentials, none of the stimuli are large enough to reach the threshold for muscle contraction. If the period of disuse without loading or tension becomes chronic, the muscles distal to the lesion begin to atrophy and lose functionality. CNS: central nervous system.

This review focuses on muscle atrophy as a detrimental secondary complication after SCI and the search for the best treatment to preserve muscle functionality. Muscle fibers function in movement through the initiation of force and power created by actin and myosin through the sliding filament theory [21]. Following SCI, there is a decrease in mechanical loading of the joints, disrupting the neuronal–muscular structural unit needed for contraction [16,20,22]. In animal models, a relatively short window of time exists for administering physical activity to prevent further atrophy. Therefore, physical activity promotes muscular hypertrophy [23]. Skeletal muscle (about 40% of total human body mass) is also an endocrine organ which secretes myokines (peptides, growth factors, and cytokines) that influence homeostasis [9,21,24]. Humans paralyzed with complete SCI endure 20–55% loss of musculature, while incomplete SCI patients endure approximately 20–30% losses, leading to a disruption of the body’s balance [4]. Atrophy results from the combination of decreased muscle protein expression and increased activity of intramuscular proteolytic enzymes including calcium-activated proteases [25]. These proteases are active in mitochondria, which participate in reactive oxygen species (ROS) generation, cellular apoptosis, and ATP synthesis [11,18,26,27]. As muscle begins to atrophy following SCI, the number of mitochondria lost appears to correlate with the increase in ROS production and a decrease in oxidative capacity [19]. This appears simultaneously with fiber type transformation from type I and type IIa to type IIx (Figure 1).

Atrophying of the musculature leads to disruption of the central nervous system (CNS). Skeletal muscle miscommunication also increases the risk of developing metabolic disorders and obesity [10,22]. The corticospinal tract (CST) is involved in executing complex movements and continues to develop beyond infancy, with new learned movements [28]. Rubro-olivary projections work in conjunction with the CST as fundamental components of recovery; if SCI disrupts both the rubrospinal and CST, motor recovery is typically prolonged.

Studies suggest that adult mammalian CNS neurons experience difficulty with the regeneration of axons after injury due to three factors: (1) the inhospitable environment of injured tissue producing inhibitors for axon sprouting/growth cones; (2) death of neurons that sustain axotomy in SCI; (3) lack of regeneration gene expression, such as GAP-43 [29]. These three factors can increase complications following SCI. The rubrospinal tract is also of particular concern following SCI. If the tract or its respective neurons are damaged in the injury process, then primary locomotion function can be seriously compromised [29]. The rubrospinal neurons have been demonstrated in animal models to increase the expression of mRNAs that code for total tubulin, T-γ1-tubulin, actin, and GAP-43 in the cervical spine 7 days post-injury. However, this expression is reduced by 50% compared to controls 14 days post-injury [29]. Rubrospinal neurons are able to regenerate in cervical transplants; this is not robustly observed in thoracic spinal cord levels. Brain-derived neurotrophic factor (BDNF) provides protection of rubrospinal neurons from atrophy [29,30]. Likewise, skeletal muscle cells release factors that support and protect motor neuron survival and neurite sprouting following denervation [31].

Cholinergic neurons also play a role in the innervation and denervation of skeletal muscle after injury. Acetylcholine (Ach), a neurotransmitter in both the central and peripheral nervous systems, is released from the cholinergic synapse of the neuromuscular junction (NMJ) by way of the action potential traveling along the axon when entering the axon terminal, stimulating neurotransmitter release and subsequent depolarization of the muscle fibers [32,33]. Normal/uninjured motor neurons and NMJ generate sufficient amounts of Ach, promoting innervation and functionality of motor neurons. Thus, due to the location and fiber type of the soleus muscle (Type I and Type IIa, below lesion), the soleus muscle has low sensitivity to Ach after SCI, in contrast to extensor digitorum longus (Type IIx) [32].

### Muscle Fiber Transformation

Alongside damage caused by muscular atrophy, there are also visible alterations to the fiber types themselves. The transformation of muscle fiber types is observed 4–7 months after SCI and reaches steady-state at 20–70 months in humans [3]. There is a definitive increase in type IIb (highly glycolytic) muscle fibers after SCI with reductions in type I (oxidative) and type IIa (mixed) muscle fibers, contributing to the increased fatigability of these muscles [22,24]. An increase in adipose tissue is observed in individuals 6 weeks post-injury, compared to able-bodied individuals of corresponding age and sex, continues over the following three months, increasing the likelihood of developing obesity [22,24,34].

**Figure 2:**
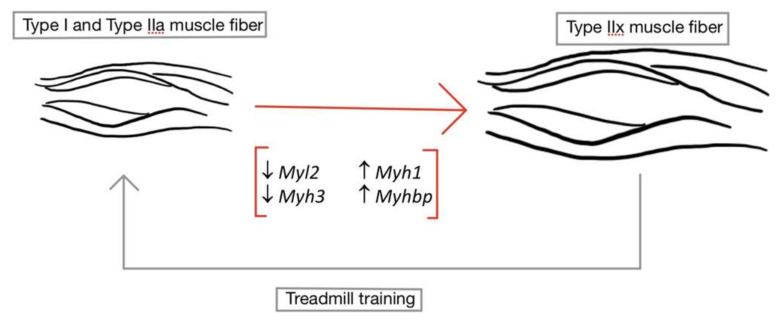
Transformation of type I and type IIa muscle fibers to type IIx muscle fibers. After SCI, type I (slow twitch, oxidative) and type IIa (fast twist, mixed) muscle fiber types undergo a morphological change into type IIx muscle fibers. This morphological change can be verified by an increase in Type IIx muscle fiber genes (*Myh1 and Myhbp*) and a reduction in type I and type IIa genes (*Myl2 and Myh3*). Therefore, unloading following injury increases the fatigability of these muscles. However, these processes may be reversed by the introduction of treadmill training, allowing the oxidative muscle fibers to regain characteristic function. (↑ signifies upregulation of genes; ↓ signifies downregulation of genes).

The rapid transition to highly glycolytic muscle fibers occurs soon after disuse and the absence of contractile forces following injury in rat models [4]. The transition to highly glycolytic muscle fibers was determined by increased gene expression of myosin heavy/light chain proteins, leading to a subsequent decrease in the oxidative fiber type (*Myl2*; myosin light chain 2 *and Myh3*; myosin heavy chain 3), with an increase in the glycolytic (*Myh1*; myosin heavy chain 1, *Myhbp*; myosin heavy chain-binding protein) during disuse. Similar expression patterns are observed in healthy individuals with the addition of resistance training in exercise regime, notably absent in SCI individuals. The addition of treadmill training in animal models has been shown to reverse the effects of fiber type transformation following SCI (with expression of *Myh1* genes) as early as 2 days post injury, as shown in Figure 2 [4]. An increase in muscular mass was found after 5 days of treadmill training for 20 min a day. Mitochondrial function and protein ubiquitination were also partially restored with the treadmill rehabilitation training, demonstrating the importance of physical exercise as a therapeutic modality [4].

## 3. Molecular Pathways and Biomarkers after SCI

**Figure 3:**
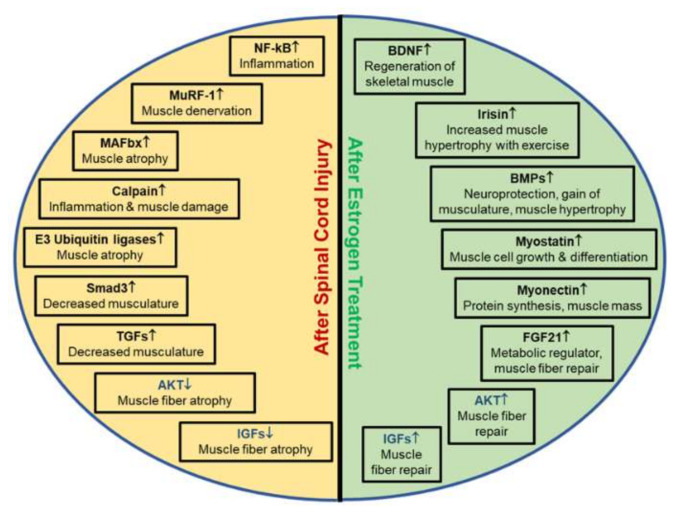
Biomarkers and their characteristics associated with muscle atrophy following SCI. Depending on the injury or treatment, biomarkers are differentially expressed, as indicated by arrows. Each unique biomarker influences inflammation, muscle hypertrophy, muscle rebuilding, muscle atrophy and/or muscle denervation in injuries. Understanding the importance of these biomarkers in SCI is critical to determine if therapeutic treatments for recovery of muscle atrophy are successful. (↑ indicates upregulation of genes; ↓ indicates downregulation of genes.)

The determination of various tissue-specific biomarkers may represent inflammation, muscle atrophy, or muscle fiber transformation occurring in myocytes, allowing for better understanding of the cascade of events following SCI, as depicted in Figure 3. For instance, nine known myokines (myocyte released cytokines) are released upon muscular contraction. These include: IL-6, IL-15, BDNF, secreted proteins which are acidic and rich in cysteine (SPARC), fibroblast growth factor 21 (FGF21), decorin, myonectin, myostatin, and irisin [21]. Each myokine has the ability to modulate cellular growth, which can lead to muscle atrophy, hypertrophy or the transition of fiber types.

### 3.1. Muscle Hypertrophy Biomarkers

While different biomarkers are expressed in muscle hypertrophy, some biomarkers encourage muscular growth, which is important for individuals recovering from SCI (Figure 3). The increased presence of IL-6, BDNF, mTOR, and irisin is detected in muscle hypertrophy following physical activity, due to protein synthesis. Irisin and BDNF also similarly upregulate muscle satellite cells after injury [18,21,29,30,35]. Moreover, fibroblast growth factor 21 (FGF21) is responsible for the activation of many intracellular pathways including signaling transducer and activator of transcription (STAT), phosphatidylinositol 3-kinase (PI3K), mitogen activation protein kinase (MAPK), and phosphoinositide phospholipase C (PLC) [3,21,36]. Furthermore, myonectin promotes the uptake of fatty acids through elevation of fatty acid transport genes, while decorin downregulates the expression of atrogin-1 and MuRF-1 [21,37,38]. Other known muscle regulators that increase with skeletal muscle activity include *Fst, Jun, Bmpr2, Actr2b, and Smad3* [4]. Thus, increased secretion of biomarkers may have dual roles in SCI, and targeting specific biomarkers could allow for improvements in functional recovery for chronic SCI.

### 3.2. Inflammatory Biomarkers

To determine whether the impact inflammation has on the body after injury, it is best to decipher which biomarkers will increase expression at or below lesion site, as well as within the muscle. SCI related inflammatory response is associated with transcriptional upregulation of various genes including *Hmox1, Ccl11, Ccr1, Lcn2, Tlr4, Ccl2, and Ccr2* which participates in neurological repair after SCI [15,39]. Similar to IL-6 released in the blood after physical activity, IL-18’s pro-inflammatory response from microglia may aggravate the lesion volume [40]. In addition, increased skeletal muscle IFN-γ, a known biomarker for inflammation in the cell, promotes apoptotic changes, stimulates inflammatory pathways, and increases the expression of caspase-3 and calcium-activated neutral proteinase (calpain). The resulting muscle fiber damage due to increased inflammation is associated with increasing NF-κB: I-κB ratios, further promoting inflammation and induction of myocyte death [41]. A recent study found that restricting the knockdown of Cx43/Cx45 hemichannels generated a decrease in muscle deterioration, as well as a decrease in NF-κB expression [18]. This attenuated inflammatory response suggests Cx hemichannels may be employed in combination with future therapeutic agents to prevent further muscle atrophy.

### 3.3. Muscle Atrophy Biomarkers

As discussed previously, the inflammatory process after injury can lead to the degeneration of muscle tissue and the eventual loss of overall motor function. SCI individuals experience persistent bed rest and decreased tension on the joints and muscles. This reduction in tension-producing activity (unloading/de-loading) is correlated with a decline in protein synthesis with eventual atrophy of the muscle [42,43]. Moreover, the ubiquitin-proteasome system (UPS) participates in degradation of multiple intracellular proteins, contributing to the catabolism of skeletal muscle. UPS-related biomarkers that signal muscle atrophy include: muscle ring finger protein 1(MuRF-1), muscle atrophy F-box protein (MAF-bx/atrogen-1), and E3 ubiquitin ligases [39,43,44]. Increased calpain expression and activity and mitochondrial damage after SCI can also lead to ubiquitination of proteins, cell death, and further muscle breakdown [18,19,27]. The associated mitochondrial dysfunction after SCI causes a cascade of reactions including the increased expression of 5’ AMP-activated protein kinase (*AMPK*), forkhead box O3 (*FOXO3*), muscle atrophy F-box gene (*MAF-bx*), muscle ring-finger protein-1 (*MuRF-1*), light chain 3(*LC3*), and BCL-2 interacting protein-3 (*Bnip3*) [18,44]. Thus, strategies for preventing mitochondrial dysfunction (and associated metabolic changes) to reduce muscle loss and improve muscle contraction may be helpful in muscular remodeling after chronic SCI.

Finally, myostatin (inhibited by decorin) also promotes muscle atrophy by preventing myogenesis [21]. Serum myostatin levels in healthy individuals are demonstrated to decrease following exercise; however, myostatin is still elevated after SCI. Myostatin knockout mice also demonstrate significantly increased muscle mass, suggesting an important role in future research in muscular atrophy following SCI [37,39].

### 3.4. Calcium-Activated Proteases

The immediate increase in intracellular free Ca^2+^ at the lesion site after injury prompts hyperactivation of calpain, which can also degrade the endogenous calpain-specific inhibitor, calpastatin [19,39,41,45]. Ubiquitous calpain occurs in two isoforms, m-calpain, and µ-calpain, which require μM and mM concentrations of Ca^2+^, respectively, for activation. Calpain activates the pro-apoptotic protein Bax and also cleaves the anti-apoptotic Bcl-xL protein into a smaller substrate for apoptotic promotion within the cell [39]. Increasing the calpain activity is directly correlated with the degradation of calpastatin and calcineurin [46,47]. Caspase-3 can also cleave calpastatin, allowing for excess calpain activity within the skeletal muscle as well as the lesion site [39,48,49,50].

Inhibitors of calpain and caspase-3 prevent cytoskeletal protein degradation following SCI [45]. Studies also demonstrate that upregulated μ-calpain activity is associated with unloading-induced muscular atrophy, thus demonstrating the influence of calpain regulation in the attenuation of inflammation after SCI [36]. A muscle-specific calpain isoform (calpain-3) has also been reported where *CAPN3* gene mutation has been implicated in limb-girdle muscular dystrophy in humans [49,51].

## 4. Treatment Paradigms to Mitigate Muscle Atrophy

### 4.1. Clinical Rehabilitation

Many injured individuals with acute SCI develop both motor and functional impairments, which can lead to difficulty completing activities of daily living and a reduction in their quality of life [52]. Rehabilitation after SCI is subdivided into three categories to determine the course of treatment: acute, subacute and chronic. The acute and subacute phases occur during the first year following injury, in which the normal neurological recovery occurs. The goal of rehabilitation during this time is prevention of further damage or complications from the injury, while enhancing long-term functionality and maintenance of the nervous system. The chronic phase incorporates assistive training after the initial 1–2 years post-injury when functional recovery subsides. Several factors can affect the extent of functional recovery after SCI including age, workers compensation claims, educational background, and the time period between injury and rehabilitation [52].

The Initiative on Methods, Measurement and Pain Assessment in Clinical Trials (IMMPACT) is used to help determine the impact of pain in SCI patients. The scale includes six criteria: (1) the affective and sensory components of pain; (2) physical functioning; (3) participant rankings of global improvement and satisfaction with treatment; (4) emotional characteristics; (5) symptoms and adverse events; (6) participant disposition. To classify pain after SCI, a three-tier system was also developed for determining the intensity of pain and how to proceed with the treatment. Tier I incorporates the musculoskeletal/visceral nociceptor and neuropathic categories. Tier II determines pain intensity levels, and Tier III strives to resolve the underlying pathology of the pain [52,53].

### 4.2. Muscle Stimulation

In combination with clinical rehabilitation, it is critical to stimulate the skeletal muscles affected by the injury in order to minimize muscle loss. A range of motion exercises have also helped to reduce pain related to muscle spasms and contractures following SCI. In a healthy individual, α-motoneurons and Ia inhibitory interneurons participate in voluntary muscle activation, leading to a harmonized contraction of agonists and relaxation of the antagonist muscle groups [35,54,55]. Unlike affected muscles in SCI patients, the majority of healthy individuals have the ability to stimulate their muscles maximally during maximum voluntary contractions [56]. Thus, involuntary isometric muscle contractions are promoted for SCI individuals through functional electrical stimulation (FES), allowing maximal contraction in affected muscles while decreasing the risk of deep vein thromboses [22,52,53]. To measure the muscular strength and associated peripheral nerve stimulation of affected muscles, assessments of maximum-voluntary contraction force of the weakened muscles can be compared to the above-lesion muscles and CNS stimulatory signals [56].

Exercise via stimulation has also been demonstrated to improve conditions associated with impairment circulation after injury. Consistent with the reduced leg muscle volume, a 25% reduction in femoral artery size can be measured following SCI with increased blood flow dilation response [17]. This reaction is a response to nitric oxide production from stress stimuli, and tissues become acclimated to this change in femoral artery size in as little as 3 weeks after injury. Thus, FES and short whole-body vibrations have been employed to increase peripheral blood flow, demonstrating the concern to decrease the amount of muscle loss after SCI.

Individuals with paraplegia often develop elevated blood pressure, hyperlipidemia, increased inflammation, and carbohydrate metabolism disorders [17]. Physical activity can increase the secretion of IL-6, leading to muscular hypertrophy and, thus, reduction in muscular atrophy [17,21]. The incorporation of activity-based physical therapy (ABPT) has been effective in psychological wellness and improved overall health after SCI [57,58]. Studies have found ABPT to be inconclusive as a stand-alone treatment for severe SCI; however, when combined with ursolic acid (an anabolic steroid injection), muscle atrophy is attenuated [58].

### 4.3. Ursolic Acid

The use of steroids in the acute SCI setting has dramatically decreased in recent years as more recent data demonstrated the lack of efficacy [19,58]. However, ursolic acid, an anabolic compound, injected intraperitonially once per day, has been used to attenuate muscular atrophy and promote signaling pathways for protein synthesis [59]. Animal models show that the administration of ursolic acid decreases atrophy by >50% in the first seven days after injury [58]. A combination of ursolic acid administration and physical activity allows for a greater increase in muscle mass, while decreasing the expression of MuRF-1 and MAF-bx proteins, as well as causing a reduction in visceral adipose tissue [58].

### 4.4. Acetoside Injection

Another common treatment used to reduce muscle atrophy is acetoside injected intramuscularly. Cultured skeletal myocytes treated with acetoside exhibit cell proliferation with increased axonal growth when compared to controls. Moreover, acetoside-treated mice achieve improved locomotor scores at 31, 55, and 62 days post-injury [9]. The bicep femoris and tibialis anterior were tested following intramuscular injection, and the overall mass of each muscle was measured following treatment. The acetoside-treated muscles were significantly larger in mass compared with vehicle treatment, suggesting that acetoside aids in the recovery and regeneration of skeletal muscle, potentially independent of the exercise-induced secretion of myokines [9,21].

### 4.5. Pyruvate Kinase Muscle Isoform 2 (PKM2)

One reaction to acetoside injection is an increase in PKM2 secretion from muscle cells [9]. Studies using recombinant PKM2 (doses of 1 and 10 ng/mL) added to cultured mouse myocytes for 3 days, showed a significant increase in cortical neuron axonal density compared to controls [9]. PKM2 is also found to promote axonal growth in the presence of CSPG, an axonal growth inhibitor secreted by astrocytes in SCI lesions [9]. The effects of acetoside are also expected to be regulated by PKM2 and the two can be used to treat chronic SCI individuals.

### 4.6. Effects of IFN-γ and Calpeptin

IFN-γ is a known pro-inflammatory cytokine which promotes apoptosis in and around the lesion site of spinal cord and damaged muscle, through (among other mechanisms) calpain activation and increased Bax:Bcl-2 ratio [39]. IFN-γ treatments have shown to cause apoptosis in rat L6 myoblast muscle cell lines, with associated cell shrinkage and membrane blebbing. Increased expression of caspase-12, caspase-3, and m-calpain can be observed in these cells [39]. Damage to mitochondria and sarcolemma also contribute to the influx of intracellular Ca^2+^ and increased calpain activation [19]. The administration of a calpain inhibitor, calpeptin, was found to significantly decrease cellular apoptosis associated with IFN-γ in L6 myoblast cells [39]; however, these inhibitors have not been approved for clinical use in SCI patients, since calpain participates in multiple other cellular growth and metabolic functions throughout the body.

### 4.7. Estrogen Effects

Estrogen is a broadly active steroid hormone with both anti-oxidant and anti-apoptotic effects [7,19,42,60,61]. Estrogen also has the ability to influence the secretion of Ca^2+^ and hinder inflammatory processes. Thus, estrogen therapy has contributed to improvements in neural protection, neuronal regeneration, and further muscular strength following menopause [7,40,61,62,63]. Improved functionality has also been observed in women compared to men after SCI, suggesting the importance of estrogen in the recovery process [19]. Due to estrogen’s protective nature against oxidative stress, glutamate toxicity, and excess cellular calcium influx, estrogen replacement therapy has also been studied in the prevention of numerous neurodegenerative diseases [64]. Estrogen also promotes angiogenesis following SCI [65,66]. Nevertheless, precautions must be taken in the clinical setting, since dosage-dependent sequalae including hypercoagulability and oncogenesis limit estrogen use in SCI patients [67,68,69,70].

Sribnick and colleagues administered estrogen as a therapeutic agent in SCI models to decrease apoptosis and prevent further intracellular Ca^2+^ release and mitochondrial damage [35,60,71]. Estrogen prevented mitochondrial cytochrome c release and calpain expression after SCI. Similar studies using estrogen have also shown decreased expression of COX-2, NF-κB, and caspase-3 [8,65]. Decreased expression of such markers suggests estrogen’s ability to decrease inflammatory responses. This reduction in neuronal cell death, inflammation, and axonal degeneration was coupled with enhanced locomotor function recovery, again providing evidence of the hormone being used for recovery.

Estrogen also plays a role in remodeling the extracellular matrix and muscle fiber expansion after disuse/unloading [42]. A study using female rats found that loss of estrogen does not halt muscle recovery, but the time for return of muscle fiber function was prolonged [42]. Recent studies have demonstrated that doses of 10–200 µg of estrogen in animal models are beneficial after SCI as neuroprotective agents [65,72,73]. This dosage protected the penumbra caudal to the injury site (preventing apoptosis of sensory and motor neurons), while keeping the dose low enough to avoid harmful side effects. Another study determined that a dosage of 4 mg/kg given twice over 24 h after SCI boosted recovery, with both anti-inflammatory and anti-apoptotic effects. The rats demonstrated visible improvements in locomotor functionality 3 weeks after SCI [19]. These dosages also demonstrated decreased expression of calpain/caspases, while preventing the astrogliosis and invasion of peripheral monocytes/neutrophils in both white and gray matter [19,40,64]. Our recent study indicates that nanoparticle-mediated delivery of E2 (5.0 µg) successfully improved locomotor function by attenuating inflammation and other secondary injury factors [72,74]. Thus, low doses of intravenous estrogen can provide multiple neuroprotective effects following SCI [19,60,64].

## 5. Conclusions

As the number of individuals suffering from SCI increases annually, the search for therapeutic modalities to improve their quality of life continues. Disconnection of the musculoskeletal system from the CNS leads to chronic unloading of the muscles and associated muscle atrophy. A number of studies have discovered strategies to help reverse muscle atrophy using pharmacological treatments in conjunction with physical therapy and muscle stimulation. In addition, the evolving use of biomarkers of inflammation, muscular atrophy, hypertrophy, and morphological fiber changes will thus further direct future research.
